# Supplementary material for: Fetal malnutrition and associated factors among term newborn babies in Jimma Zone public hospitals, South West Ethiopia
Source: PLoS One. 2025 Sep 18;20(9):e0332596. doi: 10.1371/journal.pone.0332596 (PMC12445542; doi:10.1371/journal.pone.0332596)
Supplement: Attachment — Submitted filename: Response to reviewers (1).docx [file pone.0332596.s003.docx]

## Manuscript PONE-D-24-57243

## Response to academic editors and reviewers

First of all I would like to thank you for giving us the opportunity to submit a revised draft of the manuscript“Fetal malnutrition and associated factors among term newborn babies in Jimma Zone Public Hospitals, South West Ethiopia” we appreciate the time and effort that you and the reviewers dedicated to providing feedback on our manuscript and are grateful for the insightful comments on and valuable improvement to our paper. We have incorporated most of the suggestions made by the academic editors and reviewers. Those changes are highlighted in the revised manuscript with track changes. Please see below, in blue, for a point- by-point response to the academic editors and reviewers comments and concerns.

Academic Editor1.

- Data availability statement

**Authors’ response:** - all the data are fully available without restriction and it is within the manuscript and its supporting information

- Use of English language is poor in certain sections and would require a detailed revision.
- Line 15: Thus, this study aimed to assess predictor……

**Authors’ response**: Thus, this study aimed to assess prevalence and associated factors of fetal malnutrition among term newborn babies in Jimma Zone Public Hospitals. (Found in line 17&18)

- Lines 17 and 18

**Authors’ response**: A cross-sectional study was carried out among 449-term newborns using systematic sampling techniques in Jimma Zone Public Hospitals from April 1, 2024 to July 30, 2024 (found in line 20&21)

- In the abstract and result, the absolute number (numerator and denominator) is needed together with the percentage. For example, A/B (C %)

**Authors’ response**: Thank you for your feedback we incorporate these comments in the revised manuscript

- Line 72: Materials and methods

**Authors’ response**: Materials and Methods (found in line 82)

- Line 80: April 1, 2024 to July 30, 2024

**Authors’ response**: April 1, 2024 to July 30, 2024 (found in line 90&91.

- Line 222: (Table 1); remove the word ‘see’; similarly, make a correction for other table and figure citations.
- Table 6: Follow appropriate scientific notations, for example: this, .924(.315 -2.712) should be written as, 0.924(0.315-2.712)

**Authors’ response**: Thank you for your valuable feedback. We have made the necessary corrections based on your suggestions and have reflected them in the revised manuscript

- Add the following to the declaration section: **Ethical approval and consent to participate, Consent for publication, Data availability statement, Competing interest and Funding statement**

**Authors’ response**: Thank you for your valuable feedback. We have add those parts in the declaration section in the revised manuscript

Response to reviewer

Reviewer 1

- Title: the word “predictors" is not favorable since it was cross-sectional, better to replace with "associated factors

**Authors’ response**: Dear Reviewer, thank you for your valuable comments and feedback. As per your suggestion, we have replaced the term "predictors" with "associated factors" in the title. Fetal malnutrition and associated factors among term newborn babies in Jimma Zone Public Hospitals, South West Ethiopia

- Abstract: please state separately methods of data collection for maternal data and newborn data (CAN score). Please say p-value less than 0.05. Delete “finally". Please include number of male and female newborns in parentheses.

**Authors’ response**: Under abstract part we add the method of data collection for maternal data is face-to-face interviews and reviewing medical records using semi-structured interviewer administered questionnaire and newborn data is collected by physical observation of fetal nutritional status using standardized tool (CAN score).

- We add number of female and male newborn in the parentheses, among delivered newborns 220 (49%) were males and 229 (51%) were females
- Please rewrite your conclusion. No need of this study, previous study? Please state your major findings in two or three statements. Even your recommendation is beyond the findings of your study. Please get English language proficiency from native speak

**Authors’ response**: **Conclusion**: The prevalence of fetal malnutrition in this study indicates one in five delivered newborn. Newborns with low birth weight, low placental weight, and mothers having anemia, intimate partner violence (IPV), antenatal depression, teenage pregnancy, malaria, infection, and complications during pregnancy were a strong association with fetal malnutrition. Therefore, this study recommended that all concerned bodies should be engaged in mitigate intimate partner violence, prevent infections during pregnancy, enhance maternal nutrition counseling, and address the issue of teenage pregnancy.

- Introduction: please revise your introduction particularly the coherence and grammar including punctuations. Dear authors even you missed some published studies, try to include all existing evidences to give best information to the readers.

**Authors’ response**: We appreciate your comments on the introduction section. We have made efforts to improve the grammar and coherence in the revised manuscript accordingly.

- Methods: dear authors, would you explain why you extend the age of newborns up to 48 hours? Why not within 24hours? Please give your suit why 24 - 48 hours post-partum?

**Authors’ response**: The reason for extending the age of the newborn up to 48 hours was to ensure the inclusion of mothers who delivered via cesarean section, allowing sufficient time for them to stabilize before obtaining their medical history in a comfortable and appropriate condition.

- Sample size - why you take 0.04?

**Authors’ response**: We used a 4% margin of error based on previous research findings confidence interval. In determining the sample size, we considered the p-values and confidence intervals reported in earlier studies. This margin of error was chosen to enhance the precision and reliability of the study results. Selecting an appropriate margin of error is essential for ensuring the accuracy and validity of our statistical estimates.

Who collects the CAN score data? Does the data collectors get CAN score training? Who gave the training?

**Authors’ response**: The newborn data were collected by BSc midwives under the supervision of MSc midwives who has work experiences in neonatal side. Prior to data collection, the principal investigators provided training on the use of the CAN score, including guidance on how to assess and assign scores based on the loss of fat and muscle mass across various physical characteristics of the newborn

- Intimate partner violence and low placental weight needs operational definition in the method section

**Authors’ response**: Dear academic editors, Thank you for your valuable feedback in operational definition of these variables, in the revised manuscript we put the operational definitions accordingly

How do you classify the placental weight? Have you record the morphology type?

**Authors’ response**: We classified the placenta after measuring its weight using newborn weighting scale measurement, thickness, and length by using tape meter. The classification was based on placental weight, distinguishing between low placental weight and normal placental weight, following the criteria established by previous research studies, as referenced in the manuscript

- I am not clear with your independent variables, please clear and simple. Rather it is important to move to the operational definition section.

**Authors’ response**: Our independent variables include various maternal characteristics, categorized into the following factors: socio-demographic factors, maternal nutritional and behavioral factors, obstetric factors, medical factors, psychosocial factors, and fetal factors. The fetal factors include the weight of the newborn, sex, weight of the placenta, and gestational age. We have listed the specific variables under each category.

- Result: In your logistic regression table please write only the exact p- values.

**Authors’ response**: We appreciate your comment regarding the logistic regression table. Accordingly, we have made the necessary revisions as per your suggestion
