# Supplementary material for: Fetal malnutrition and associated factors among term newborn babies in Jimma Zone public hospitals, South West Ethiopia
Source: PLoS One. 2025 Sep 18;20(9):e0332596. doi: 10.1371/journal.pone.0332596 (PMC12445542; doi:10.1371/journal.pone.0332596)
Supplement: Attachment — Submitted filename: Response to reviewers.docx [file pone.0332596.s004.docx]

## Manuscript PONE-D-24-57243

## Response to academic editors and reviewers

First of all I would like to thank you for giving us the opportunity to submit a revised draft of the manuscript “Fetal malnutrition and associated factors among term newborn babies in Jimma Zone Public Hospitals, South West Ethiopia” we appreciate the time and effort that you and the reviewers dedicated to providing feedback on our manuscript and are grateful for the insightful comments on and valuable improvement to our paper. We have incorporated most of the suggestions made by the academic editors and reviewers. Those changes are highlighted in the revised manuscript with track changes. Please see below, in blue, for a point- by-point response to the academic editors and reviewers comments and concerns.

**Academic Editor1.**

Table 6: P=0.000, what does it mean. The author should make it meaningful.

**Authors’ response: -** Thank you for your valuable feedback. We agree with your point, and in the revised manuscript we have approximated this number to 0.001 for clarity and consistency

**Table 6: Why you consider as a reference age group from 25-34? Do you have reason?**

**Authors’ response: -** Thank you for raising this important point. The comment is correct, and we agree with the observation. The reason for considering the age group 25–34 years as the reference category is that it represents the biological optimum and lowest-risk reproductive age group. Women in this age bracket are often at their peak reproductive potential, with high fertility and relatively lower risks of pregnancy complications compared to younger mothers (<20 years) and older mothers (≥35 years).

For studying risk factors such as fetal malnutrition, it is necessary to identify a ‘baseline’ group for comparison. The 25–34 age range provides this baseline because it reflects the most physiologically stable and nutritionally favorable stage, thereby minimizing the influence of age-related risks. Furthermore, evidence from epidemiological studies consistently demonstrates a U-shaped relationship between maternal age and adverse outcomes: increased risks at the extremes (<20 and ≥35 years), with the lowest risk observed in the middle group (25–34 years). For these reasons, this age group is commonly used as the reference category in similar studies.

**The denominator still should be included in the percentage of your both in the abstract and main result**

**Authors’ response: - Thank you for this valuable comment. In the revised manuscript, we have included both the denominators and the corresponding percentages in the relevant sections.**

**Some of the tables can be integrated and prepared. Think of it.**

**Authors’ response: -** Thank you for your observation. Yes, the tables have been integrated. Specifically, we have combined Table 3 and Table 4, which presented maternal obstetric and medical factors, into a single table in the revised manuscript

**If the reviewer comments include a recommendation to cite specific previously published works, please review and evaluate these publications to determine whether they are relevant and should be cited**

**Authors’ response: -**Thank you for this constructive comment. Although the reviewer did not recommend specific previously published works, the suggestion to check for uncited relevant literature was very helpful. We carefully reviewed the existing body of evidence, and most of the relevant studies have now been incorporated

**Please review your reference list to ensure that it is complete and correct. If you have cited papers that have been retracted, please include the rationale for doing so in the manuscript text, or remove these references and replace them with relevant current references. Any changes to the reference list should be mentioned in the rebuttal letter that accompanies your revised manuscript.**

**Authors’ response: -** Thank you for your observation. We have carefully reviewed the entire reference list to ensure its completeness and accuracy, and the necessary corrections have been made. These updates have been highlighted in the revised manuscript.
